# Association between self-rated health and mortality: 10 years follow-up to the *Pró-Saúde* cohort study

**DOI:** 10.1186/1471-2458-12-676

**Published:** 2012-08-20

**Authors:** Joanna Miguez Nery Guimarães, Dóra Chor, Guilherme Loureiro Werneck, Marilia Sá Carvalho, Claudia Medina Coeli, Claudia S Lopes, Eduardo Faerstein

**Affiliations:** 1 Department of Epidemiology, National School of Public Health, Oswaldo Cruz Foundation, R Leopoldo Bulhões 1480, Rio de Janeiro, RJ, 21041-210, Brazil; 2 Department of Epidemiology, Institute of Social Medicine, State University of Rio de Janeiro, R São Francisco Xavier 524, Rio de Janeiro, RJ, 20550-900, Brazil; 3 Scientific Computing Program - PROCC, Oswaldo Cruz Foundation, Av Brasil 4365, Rio de Janeiro, RJ, 21045-900, Brazil; 4 Institute of Public Health Studies, Federal University of Rio de Janeiro, Av Horácio Macedo S/N, Rio de Janeiro, RJ, 21941-568, Brazil

## Abstract

**Background:**

The association between self-rated health (SRH) and mortality is well documented in the literature, but studies on the subject among young adults in Latin America are rare, as are those evaluating this association using repeated SRH measures, beyond the baseline measurement. This study aims to evaluate the association between SRH evaluated at three data collection stages and mortality.

**Methods:**

Cox regression models were used to examine the association between SRH (Very good, Good, Fair/Poor) varying over time and mortality, over a 10 year period, in a cohort of non-faculty civil servants at a public university in Rio de Janeiro, Brazil (*Pró-Saúde* Study, n = 4009, men = 44.4%).

**Results:**

About 40% of the population changed their self-rating over the course of follow-up. After adjustment for self-reported physician-diagnosed chronic diseases and other covariates, men who reported “Fair/Poor” SRH showed relative hazard of death of 2.13 (CI95% 1.03-4.40) and women, 3.43 (CI95% 1.23-9.59), as compared with those who reported “Very good” SRH.

**Conclusions:**

In a population of young adults, our findings reinforce the role of SRH as a predictor of mortality, even controlling for objective measures of health.

## Background

Self-rated health (SRH) is a subjective measure that is being used to monitor the health of various populations [1,2]. Although the factors taken into consideration by SRH are not yet totally understood, individual SRH seems to reflect a comprehensive perception of health which includes biological, psychological and social aspects unlikely to be grasped by external observers [1]. It can also express health behaviors, psychological and social well-being, trajectories in health over time, socio-economic conditions, and overall quality of life [3]. Symptoms such as chronic pain and fatigue are also pointed to as important elements that may influence SRH, while even sub-clinical dysfunctions unlikely to be diagnosed as medical conditions can be perceived by individuals and incorporated into their self-assessment [4].

The single item used to gauge SRH – commonly implemented by the question “In general, how would you rate your health?” – has attracted health researchers' attention because it is easy to answer [2] and low-cost [5], but especially due to the accumulated evidence of its association with morbidity [6], health service utilization [1,5], socio-demographic indicators such as age, sex and education [7] and, most importantly, mortality [8-10].

A number of studies evaluating population groups in various countries for varying follow-up periods have found that respondents who perceived themselves to be in poor health display hazard of mortality 2 to 7 times greater than those who perceived their health to be excellent, even after adjustment for diseases and other important health conditions [2,8,9]. A dose–response pattern is also observed by Likert-type scale response options (for example, Very good, Good, Fair, Poor or Very Poor), with the likelihood of death increasing gradually towards the “Very Poor” response category [8,9,11].

Although the relationship between SRH and mortality is well documented in the literature, most studies have evaluated SRH at only one point in time, generally at the study baseline [10-13]. However, SRH is expected to vary over the course of cohort follow-up [14-16], and these changes must be incorporated into the analyses, because worsening SRH is an important predictor of mortality [15].

There are many issues that give good reason for studying the association between SRH and mortality in our specific cultural context. First, there are few studies on such relationship arising from Brazil, a country with a different socioeconomic profile as compared to most developed countries where the majority of reports on the relationship between SRH and mortality comes from. The three existing studies have investigated the association only in populations over 60 years of age and used only one measure of SRH, taken at the baseline [17-19]. Second, the pattern of mortality by age and cause and the profile of response to the SRH question are quite diverse; both might per se contribute to a different pattern of association between SRH and mortality. Finally, there are recognized cultural differences in SRH classification [20], which also might alter the relationship between SRH and mortality in different contexts. The aim of this study is to evaluate the association between SRH reported at three data collection stages and mortality, over a period of 10 years, among a cohort of non-faculty civil servants at a public university in Rio de Janeiro.

## Methods

### Study design and population

This study draws on a longitudinal prospective study of non-faculty civil servants at a university in Rio de Rio de Janeiro, Brazil (the *Pró-Saúde* study), directed mainly to investigating social determinants of health outcomes.

At stage 1 of the study (1999), all regular employees in technical administrative positions were considered eligible, except those who had retired, were on leave of absence, had been dismissed or seconded to other institutions (N = 4030, 91% of the eligible population). The analyses presented here include all employees participating in stage 1 with valid responses to the question on SRH (N = 4009). Of these, 80.7% (N = 3237) were followed up at stage 2 of the study (in 2001) and 81.2% (N = 3255), at stage 3 (in 2006). These participants’ life status was monitored until May 2009. The responses for SRH at stage 2 and stage 3, whenever available, were incorporated into the analyses.

### Measurements

Participants filled out a self-administered questionnaire. Self-rated health was evaluated on an ordinal basis, as measured by the question “In comparison with people of your age, how do you rate your own overall health status?”. The response options were: “Very good”, “Good”, “Fair” or “Poor”. For the analyses, the levels “Fair” and “Poor” were grouped into a single category, because the category “Poor” was mentioned by only a small number of employees at stage 1 (N = 63). A test-retest reliability study with a two-week interval between responses was performed among individuals not enrolled in the study cohort, but whose sex, age and literacy profile was similar to that of the cohort. Reliability for the SRH reported at stage 1 of the study (1999) was estimated using weighted kappa (square weighting), returning a value of 0.65 (CI95% 0.54-0.72).

Deaths occurring in the cohort between 1999 and 2009 were identified by consulting the university human resources department. In order to investigate their causes, these deaths were located in the national Mortality Information System (SIM) database up to 2006 – the most recent data available – by means of probabilistic record linkage technique. Reclink [21] was used, applying a five-step blocking strategy with keys formed by combining sex and Soundex phonetic codes for first and last names. Records were paired by name, mother's name and date of birth, with the linkage parameters estimated by means of the EM algorithm [22]. Using this strategy, 96% of the deaths occurred up to 2006 were identified in the mortality database. It was opted to consider the information from the human resources department as a primary source with a view to: (1) permit identification of the events that occurred up to the end of the observation period (2009), since deaths occurring after 2006 were not available in the SIM; and (2) prevent identification of deaths that did not in fact occur (false positive errors). These latter, even when random, have greater impact on the validity of ratio-type measures of association than false negative errors [23]. Participants still living in May 2009 were right-censored.

The covariates included in the study were defined as: a) age (years): < 35, 35 to 44, 45 to 54 and >54; b) sex: male or female; c) income: per capita family income terciles in U.S. dollars (US$), calculated by dividing the median of the net income bracket by the number of dependents on that income, and converting to August 1999 dollar values (median date of entry into the study in 1999) of US$1.00 = R$1.70; d) schooling: fundamental (up to incomplete high school), middle (complete high school and incomplete university) or university (complete university or more); e) chronic diseases: self-reported lifetime medical diagnosis of arterial hypertension and/or diabetes mellitus and/or myocardial infarction and/or cerebral vascular accident and/or pulmonary emphysema/chronic bronchitis – categorised as none, or at least one, of these conditions; f) presence of common mental disorders: evaluated on the General Health Questionnaire (GHQ-12) scale – categorised as present (at least three affirmative responses among the 12 items of the scale) or absent [24]; g) body mass index (BMI = weight/height^2^, in kg/m^2^): calculated from weight and height measured and classified according to World Health Organization recommendations – categorised as underweight or normal weight (<25 kg/m^2^); overweight (25 to 29.9); or obesity (≥30) [25]; h) current habit of smoking: yes or no; i) marital status: married, separated, widowed or single; and j) color/race: as reported by the participant from the Brazilian census ethnic categories – white, ‘pardo’ (mulatto), black, asian or indigenous. Those who classified themselves as asian or indigenous were grouped into a single category (“others”), as they represented only 2.5% of the population.

All variables analyzed were collected at stage 1 only, except the main exposure variable (SRH) and age, updated in 2001 (stage 2) and 2006 (stage 3), and the covariate “chronic diseases”, updated in 2001 but not available in 2006. The pattern of change in the SRH over time was investigated.

All participants signed a declaration of informed consent, and the research protocols were approved by the Research Ethics Committee of the Institute of Social Medicine of the State University of Rio de Janeiro.

### Statistical analysis

The association between SRH and mortality was estimated using extended Cox proportional hazard models with both SRH and chronic diseases varying over time. Follow-up started on study entry date, and the variable was updated at each new interview. The Kaplan-Meier estimator and the log-rank test (p < 0.15) were used to select the variables to be evaluated in bivariate Cox models, where hazard ratios (HR) were estimated. The variables that proved statistically significant in the bivariate models (p < 0.05) were then included in the multivariate analysis.

Variables' entry into the multivariate models was determined on the hierarchical causality theoretical model, including first the distal characteristics (age, sex, color/race, schooling, income and marital status) and then the intermediate characteristics (chronic diseases, smoking, BMI and common mental disorders). In order to be maintained as a confounder in the multivariate model, each variable had to be associated with the outcome at a 5% level of significance (p < 0.05) and/or alter the effect of SRH on mortality by at least 20%. It was decided *a priori* that “chronic diseases” would be retained in the final model independently of these criteria, due to its importance as an alternative explanation of the causal chain. The Cox model’s assumption of proportional hazard for each variable over time was checked by analysis of Shoenfeld’s residual analysis and the functional form of the continuous variables age and income was checked by analysis of Martingale’s residual analysis (p < 0.05). All analyses were performed using the R statistical package, version 2.10.0 [26] with the survival library [27].

## Results

Due to the difference in hazard of death encountered among men and women, by SRH, the analyses were performed separately by sex (Table 1). The group studied was mostly female (55.6%) and predominantly young adults (mean age = 40.1 years) (Table 1). About 40% had completed undergraduate or postgraduate education, 52% classified themselves as white, and just over 20% were smokers at the time of the study. Approximately 30% reported at least one medical diagnosis of some disease of interest. In addition, more than half the population were overweight (BMI ≥ 25) and the prevalence of common mental disorders was estimated at more than 30%.

**Table 1:** Study population (n and %) and deaths for men and women (n and cumulative incidences - Risk), by variables analyzed, measured at stage 1. Pró-Saúde Study, 1999

**Variables**	**Total population (N = 4009)**	**Deaths (N = 117)**		P-value (log-rank*)	
	**n (%)**	**Men (N = 70)**	**Women (N = 47)**	**Men**	**Women**
		**n (Risk)**	**n (Risk)**		
**Self-rated health**					
Very good	1132 (28.2)	14 (0.026)	3 (0.005)	< 0.001	< 0.001
Good	2131 (53.2)	30 (0.031)	16 (0.014)		
Fair/Poor	746 (18.6)	26 (0.096)	28 (0.059)		
**Age**					
< 35	1121 (28.0)	2 (0.004)	4 (0.007)	< 0.001	< 0.001
35-44	1734 (43.2)	19 (0.026)	14 (0.014)		
45-54	877 (21.9)	30 (0.077)	12 (0.024)		
> 54	277 (6.9)	19 (0.176)	17 (0.101)		
**Sex**					
Female	2228 (55.6)	-	-	-	-
Male	1781 (44.4)	-	-		
**Schooling** §					
University	1604 (40.5)	13 (0.022)	7 (0.007)	< 0.001	< 0.001
Secondary	1422 (35.9)	21 (0.031)	20 (0.027)		
Fundamental	936 (23.6)	35 (0.071)	19 (0.043)		
**Per capita family income (terciles. US$)** ‡					
515–3236	1097 (29.1)	12 (0.027)	6 (0.009)	0.006	< 0.001
245–514	1615 (42.9)	18 (0.027)	15 (0.016)		
16–244	1057 (28.0)	32 (0.058)	21 (0.041)		
**Chronic disease ∞**					
No disease	2956 (73.9)	37 (0.028)	18 (0.011)	< 0.001	< 0.001
At least one	1045 (26.1)	33 (0.075)	29 (0.048)		
**Smoking** Δ					
Currently not smoking	2934 (75.9)	33 (0.026)	33 (0.020)	< 0.001	0.53
Currently smoking	932 (24.1)	36 (0.081)	12 (0.025)		
**BMI †**					
< 25	1823 (46.5)	26 (0.037)	18 (0.016)	0.23	0.02
25–29.9	1444 (36.7)	27 (0.035)	13 (0.019)		
≥ 30	662 (16.8)	16 (0.057)	15 (0.039)		
**Race** Ψ					
White	2076 (52.4)	33 (0.035)	17 (0.015)	0.62	0.05
Pardo (Mulatto)	1164 (29.4)	22 (0.039)	13 (0.021)		
Black	620 (15.7)	12 (0.055)	13 (0.032)		
Others**	100 (2.5)	2 (0.045)	3 (0.054)		
**Common Mental Disorders** Ø					
Absent	2620 (69.3)	45 (0.035)	24 (0.018)	0.23	0.39
Present	1162 (30.7)	19 (0.048)	18 (0.023)		
**Marital Status** €					
Married	2383 (61.0)	48 (0.039)	16 (0.014)	0.19	0.03
Separated	610 (15.6)	9 (0.051)	15 (0.035)		
Widowed	115 (2.9)	2 (0.118)	4 (0.041)		
Single	801 (20.5)	9 (0.027)	9 (0.019)		

*p < 0,15 (used as criterion to select variables for the Cox models).

**Asian (n = 60) and Indigenous (n = 40).

Not answered (n): § = 47, ‡ = 240, ∞ = 8, Δ = 143, † = 80, Ψ = 49, Ø = 227, € = 100.

At stage 1, health was self-rated as “Very good” by 28.2% of participants, “Good” by 53.2%, “Fair” by 17% and “Poor” by 1.6%. Self-perceived health was worse among women than among men, with 21.3% reporting SRH as “Fair” or “Poor”, compared with 13.2% of the men (p < 0.001). In both sexes, worse SRH was also observed among the older individuals, those with less income, less schooling, who were widowed, who reported some disease, had higher BMI, were classified as positive for common mental disorders or as smokers, for both sexes (p < 0.05) (data not presented). At stages 2 and 3, SRH prevalences were, respectively, 27.8% and 26.3% (Very good), 51.5% and 50.7% (Good), 17.8% and 20.2% (Fair), and 1.6% and 2.2% (Poor).

**Figure 1:**
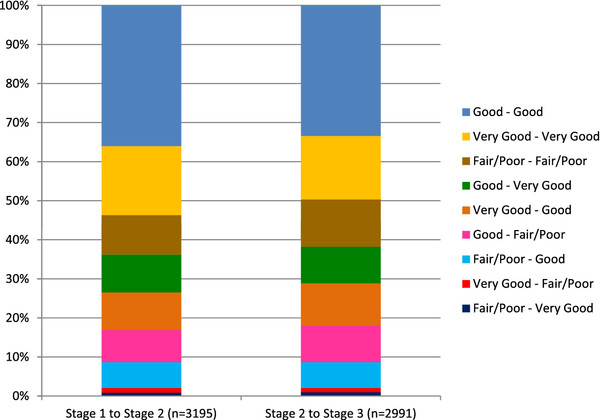
**Change in SRH category over follow-up (from stage 1 to stage 2 and from stage 2 to stage 3).** Pró-Saúde Study, 1999–2009.

As regards changes in SRH over the course of follow-up (Figure 1), 36.2% of the population changed SRH category from stage 1 to stage 2, and 38.2% from stage 2 to 3. The pattern of change was more often towards worsening health than towards improvement, and that difference was most marked from stage 2 to stage 3 (21.3% worsened and 16.9% improved) than from stage 1 to stage 2 (19.1% worsened and 17.1% improved). However, the proportion of participants whose SRH deteriorated or improved by two categories (from “Very good” to “Fair/Poor”, or the opposite) was very small: 1.2% and 0.8% from stage 1 to stage 2; and 1.1% and 1.0% from stage 2 to stage 3, respectively.

**Figure 2:**
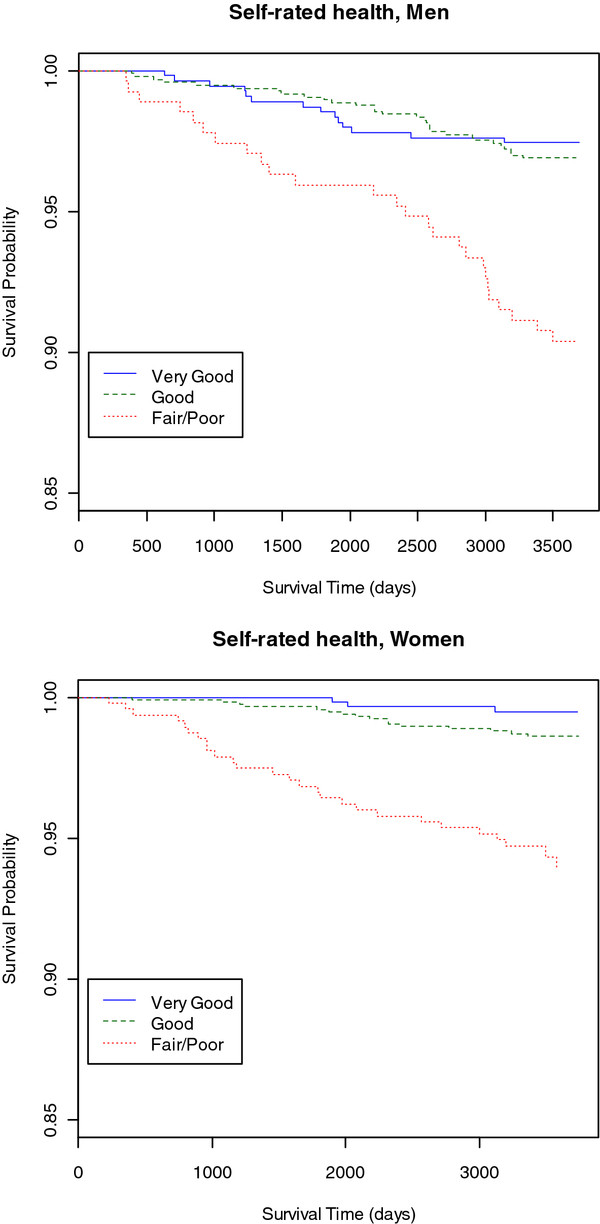
**Kaplan-Meier estimates of survival among men and women, by the variable SRH, measured at stage 1.** Pró-Saúde Study, 1999.

Of the 4009 participants with complete SRH data at stage 1 (99.5% of participants at that study stage), 117 had died by May 2009 (Table 1) and the cumulative incidence of death among the men was almost double that estimated for the women (0.039 and 0.021, respectively). For both sexes, cumulative incidence of mortality increased with worsening SRH (Table 1). Log-rank tests for survival differences were all significant, except for common mental disorders, BMI, race and marital status among men; and common mental disorders and smoking among women. Figure 2 shows the Kaplan-Meier plots illustrating the effect of SRH measured at stage 1 of the study, among men and women.

The causes of mortality for deaths occurring up to 2006 (N = 80) were distributed as follows: diseases of the circulatory system (N = 24, 30%), neoplasms (N = 13, 16%), diseases of the respiratory system (N = 11, 14%), external causes (N = 8, 10%), infectious and parasitic diseases (N = 6, 8%), endocrine, nutritional and metabolic diseases (N = 6, 8%), diseases of the digestive system (N = 4, 5%), symptoms, signs and abnormal clinical and laboratory findings, not elsewhere classified (N = 4, 5%), and others (N = 4, 1% each). Distribution of causes of mortality was similar for men and women, with the exception of external causes, which constituted the second cause of death among men and did not figure among causes of death for women.

The crude survival functions indicated that hazard of death for women with “Fair/Poor” SRH was seven times greater than for those with “Very good” SRH (Table 2). Among men, the hazard was four times greater than for the reference category. After adjustment (model 4), men who reported “Fair/Poor” SRH showed relative hazard of death of 2.13 (CI95% 1.03-4.40) and women, 3.43 (CI95% 1.23-9.59), as compared with those who reported “Very good” SRH. Hazard of death for participants with “Good” SRH was not statistically different from the hazard of those whose SRH was “Very good”. Schoenfeld’s residual analysis confirmed the proportional hazard assumption. As the Martingale’s residuals indicated that the variable “income” did not display a linear function form, it was categorized in terciles.

**Table 2:** Crude and adjusted mortality hazard ratios (HR), confidence intervals (CI95%), coefficients (betas - β) and standard errors (SE) for SRH, among men and women. Pró-Saúde Study, 1999–2009

**Cox models, Men**	HR*	**CI95%**	**β**	**SE**
**Model 1: SRH (crude)**				
Very good	1.00			
Good	1.44	0.72 - 2.88	0.36	0.35
Fair/Poor	4.45	2.23 - 8.88	1.49	0.35
**Model 2: SRH + Age**				
Very good	1.00			
Good	1.42	0.71 - 2.85	0.35	0.35
Fair/Poor	3.42	1.71 - 6.82	1.23	0.35
**Model 3: SRH + Age + Diseases**				
Very good	1.00			
Good	1.26	0.63 - 2.55	0.23	0.36
Fair/Poor	2.63	1.28 - 5.40	0.97	0.37
**Model 4: SRH + Age + Diseases + Smoking**				
Very good	1.00			
Good	1.08	0.53 - 2.18	0.07	0.36
Fair/Poor	2.13	1.03 - 4.40	0.76	0.37
				
**Cox models, Women**	HR*	**CI95%**	**β**	**SE**
**Model 1: SRH (crude)**				
Very good	1.00			
Good	1.27	0.45 - 3.60	0.24	0.53
Fair/Poor	7.04	2.72 - 18.19	1.95	0.48
**Model 2: SRH + Age**				
Very good	1.00			
Good	1.20	0.43 - 3.50	0.21	0.53
Fair/Poor	5.25	2.02 - 13.66	1.66	0.49
**Model 3: SRH + Age + Schooling**				
Very good	1.00			
Good	1.11	0.39 - 3.17	0.10	0.54
Fair/Poor	4.09	1.52 - 11.04	1.41	0.51
**Model 4: SRH + Age + Schooling + Diseases**				
Very good	1.00			
Good	1.02	0.35 - 2.94	0.02	0.54
Fair/Poor	3.43	1.23 - 9.59	1.23	0.52

*p < 0.05.

## Discussion

As far as we were able to discover, this study, which involved follow-up over a 10-year period, is the first in Brazil to investigate the association between SRH and mortality in a population of young adults. Men and women with “Fair/Poor” SRH had 2.1 and 3.4 times greater hazard of mortality, respectively, than those with “Very good” SRH, independently of reporting diagnoses of chronic diseases and other covariates.

A number of studies [4,10], including review articles [2,8] and meta-analysis [9], point to SRH as an independent predictor of mortality. In Latin America the only three studies of this subject – all evaluating populations of older adults – come from Brazil. Two of them also encountered greater hazard of mortality for individuals with worse SRH [18,19]. In the third study, excess hazard ceased to be significant after adjustment for cognitive function [17].

The prevalences of the worst category of SRH estimated in our population were similar to those found among industrial workers in Brazil [28] and lower than observed in Brazil's overall population [7,29]. The differences between our findings and those of population-based studies can be explained by the fact that our population was younger, had permanent employment and more schooling, which characterize better conditions of life and health than those of the overall population. The incidence of mortality in the cohort (0.25%) was also lower than the mortality rate observed in the population from 30 to 59 years old in Rio de Janeiro State (0.56%) (mean for the period from 1999 to 2006) [30]. The *Pró-Saúde* study cohort and the population of Rio de Janeiro State have similar profiles in terms of causes of mortality, except for external causes, which had more influence on mortality in the overall population than in the cohort, and diseases of the respiratory system, which occurred more often in the *Pró-Saúde* study population [30].

The results of previous studies have varied as regards whether hazard of death, comparing the worst and best SRH categories, is greater among men or women. Some estimated greater relative hazard of mortality among women than among men associated with “Poor” SRH [1,16,31]; others found greater relative hazard among the men [32,33]. In our study, relative hazard of mortality was higher for women than for men. The fact that external causes were the second cause of death among the men and did not figure among causes of death for women may have contributed to this result, given that these conditions are less associated with self-rated health than other causes of death [4]. However, this difference between hazard of death for men and women must be interpreted with caution in our study, because there are few observations in some categories, (e.g., only 3 women in the “Very good” SRH category had died) generating imprecise estimates (model 4, Table 2).

The presence of diseases is identified as the main potential confounder of the relationship between SRH and mortality [11]. In our study, although inclusion of this variable contributed to reducing the strength of the association between “Fair/Poor” SRH and mortality (reduction of age-adjusted HR by 32.4% and 27.3% in men and women, respectively), SRH continued to be an independent predictor of mortality. Other studies that adjusted for the presence of diseases found similar results. Mossey & Shapiro [12], in a pioneering study of the association between SRH and mortality, showed that the mortality hazard associated with worse SRH was stronger than the mortality hazard associated with objective measures of health. In the study by Idler et al. [11], SRH was a significant predictor of mortality, even when physical health status was taken into consideration. In the same way, Mackenbach et al. [13] showed that adjusting for a set of self-reported chronic diseases, for socio-demographic variables and for behavioral risk factors, attenuated the effect of SRH on mortality by about 44%, in comparison to the effect measure adjusted only for sex and age; nonetheless, the excess mortality risk associated with worse SRH continued about four times greater. Other authors, on the contrary, observed that the presence of diseases explains the absence of an association between SRH and mortality [10,16,32].

Idler & Benyamini [8] suggest some possible interpretations for the effect of SRH on mortality, independently of the presence of diseases and other risk factors. SRH is an accurate, inclusive measure able to reflect symptoms of existing diseases still at prodromal stages, or even the influence of family risk factors on health. In addition, it represents a dynamic assessment that considers health trajectories and not just the health status at the time of assessment. It is also related to behaviors that affect health status, such as lesser adhesion to preventive practices and to treatment. Moreover, it is a measure that can indicate the presence or absence of psychosocial resources capable of attenuating decline in health. Manderbacka [34] suggests that in addition to the medical model of health, adopting health promotion messages and "healthy" lifestyles are important factors contributing to health assessments.

Many studies have indicated that the ability of SRH to predict mortality diminishes with increasing cohort follow-up time [10,31,35]. This result may possibly stem in part from the use of SRH measured at the baseline alone, making it a good predictor of early mortality, but not of late mortality. The studies that have investigated the association between SRH as a time-dependent covariate and mortality using Cox regression [14-16,36,37] are not that frequent, but the results are consistent.

In the study by Strawbridge & Wallhagen [14], time-dependent SRH was a predictor of mortality among women and men from 21 to 94 years of age (relative hazard = 1.44; CI95% 1.25-1.65). Han et al. [15] investigated SRH among older women at baseline and every six months for three years. Change in SRH from “Excellent” to “Poor” entailed twice the hazard of death as compared to stable “Excellent” SRH.

Some limitations of this study deserve mention. Our results might have been biased due to lack of complete information on changes of SRH over time for the participants. About 14% of them had only the first baseline SRH measure, and other 10% had only two SRH measures (baseline plus SRH recorded on stages 2 or 3). The potential effect of such problem on the results is unknown, but one might suppose that those who drop out would probably have worse health as compared to their earliest SRH evaluation and higher probability of death. Including only the first SRH assessment for these participants would probably lead to underestimation of the strength of the association between SRH and mortality. In addition, we didn’t have the measure of chronic conditions on stage 3, thus possibly slightly overestimating the independent effect of SRH. Last, it was not possible to update the status of some covariates in our analyses. However, considering that the population is made up of staff at a single public institution, changes in income and schooling are uncommon.

Moreover, deaths occurring after 2006 could not be identified, as they were not available in the Mortality Information System (SIM). However, the high proportion (96%) of deaths recorded in the university human resource system that were also found in the SIM database between 1999 and 2006 warrants our belief that the university records for deaths occurring from 2007 to 2009 are valid. Besides, the university records system is extremely reliable, as the family must notify the institution of any death in order to secure their right to a regular pension and funeral costs. Lastly, it cannot be guaranteed that residual confounding is absent, given that only some medical diagnoses were included as self-reported chronic diseases, and objective measures of health (biochemical tests, electrocardiogram, etc.) were not used. Nonetheless, we believe that this potential residual confounding is not considerable, because a number of studies using objective measures have arrived at results similar to ours [11,12,38].

Analysis of SRH in three categories (instead of the “SRH positive”/“SRH negative” dichotomy found in most research) and also the use of both SRH and presence of diseases as time-dependent covariates, make the results of this study more robust and comparable to those of the few studies that use a similar strategy. We consider that analysis of SRH that changes over time is the most appropriate analytical method for investigating the relationship between this variable and mortality, given that these alterations are frequent. Failure to incorporate such information can result in misclassification, which in our study would affect about 40% of the participants.

As regards external validity, the results obtained in the *Pró-Saúde* study cohort may represent an approximation to what is occurring in the middle strata of the economically active population of Brazil's major metropolises. Subsequent studies could investigate the role of SRH in predicting specific causes of mortality, which was not possible in the *Pró-Saúde* study given the small number of deaths. It would also be interesting to ascertain whether specific causes of mortality can explain the differences observed between men and women in the SRH-mortality relationship. It is also suggested that studies investigate different SRH trajectories over time, and their association with mortality.

## Conclusions

Our findings reinforce the importance of using SRH in epidemiological research as a simple, low-cost and complementary measure for population health monitoring.

## Competing interests

The authors declare that they have no competing interests.

## Authors’ contributions

JMNG wrote the first draft of the manuscript. JMNG and MSC carried out the data analyses. JMNG, DC, GLW, CMC, CSL and EF were responsible for the conception and design of the study. All authors significantly contributed to the interpretation of the results, commented extensively on subsequent revisions and have read and approved the final manuscript.

## Pre-publication history

The pre-publication history for this paper can be accessed here:

http://www.biomedcentral.com/1471-2458/12/676/prepub
